# Analysis and Recognition of Traditional Chinese Medicine Pulse Based on the Hilbert-Huang Transform and Random Forest in Patients with Coronary Heart Disease

**DOI:** 10.1155/2015/895749

**Published:** 2015-06-09

**Authors:** Rui Guo, Yiqin Wang, Hanxia Yan, Jianjun Yan, Fengyin Yuan, Zhaoxia Xu, Guoping Liu, Wenjie Xu

**Affiliations:** ^1^Laboratory of Information Access and Synthesis of TCM Four Diagnosis, Shanghai Univerisity of Traditional Chinese Medicine, Shanghai 201203, China; ^2^Center for TCM Information Science and Technology, Shanghai University of Traditional Chinese Medicine, Shanghai 201203, China; ^3^Center for Mechatronics Engineering, East China University of Science and Technology, Shanghai 200237, China; ^4^The first Affiliated Hospital, School of clinical Medicine of G.D.P.U, Guangzhou 51000, China

## Abstract

*Objective*. This research provides objective and quantitative parameters of the traditional Chinese medicine (TCM) pulse conditions for distinguishing between patients with the coronary heart disease (CHD) and normal people by using the proposed classification approach based on Hilbert-Huang transform (HHT) and random forest. *Methods*. The energy and the sample entropy features were extracted by applying the HHT to TCM pulse by treating these pulse signals as time series. By using the random forest classifier, the extracted two types of features and their combination were, respectively, used as input data to establish classification model. *Results*. Statistical results showed that there were significant differences in the pulse energy and sample entropy between the CHD group and the normal group. Moreover, the energy features, sample entropy features, and their combination were inputted as pulse feature vectors; the corresponding average recognition rates were 84%, 76.35%, and 90.21%, respectively. *Conclusion*. The proposed approach could be appropriately used to analyze pulses of patients with CHD, which can lay a foundation for research on objective and quantitative criteria on disease diagnosis or Zheng differentiation.

## 1. Introduction

Traditional Chinese medicine (TCM) is an ancient medical practice system which emphasizes regulating the integrity of the human body and its interrelationship with natural environments [1]. Zheng (meaning syndrome or pattern) is a unique TCM concept. It is the overall physiological and/or pathological pattern of the human body in response to a given internal and external condition, which usually is an abstraction of internal disharmony defined by a comprehensive analysis of the clinical symptoms and signs gathered by a practitioner using inspection, auscultation, olfaction, interrogation, and palpation of the pulses [2]. Chinese practitioners diagnose diseases through “Zheng differentiation.” The Zheng differentiation of TCM considers the etiology, location, nature, and condition of a disease during a specific stage of the disease process based on clinical symptoms and signs. Pulse taking is one of the key methods to gather the signs and symptoms of patients by a practitioner. During pulse taking, TCM practitioners place their fingers on the radial artery, from which various physiological and pathological conditions can be detected. Traditional pulse taking has important clinical value on the diagnosis and prognosis of the diseases, especially angiocardiopathy. Accurate pulse taking can only be done by TCM practitioners with years of experience. Therefore, objective and quantitative pulse diagnosis is highly desirable, which is of help to establish the objective and quantitative criteria on disease diagnosis or Zheng differentiation.

Coronary heart disease (CHD) is considered a primary cause of death in developed countries and is predicted to be one of the most common causes of death worldwide by 2020 [3]. Early diagnosis and prevention of CHD are essential and have critical public health implications. Identifying, vascular lesions in early stages to reverse and prevent CHD, stroke, sudden death, and other malignant vascular events are crucial [4]. Previous studies have been conducted to clarify and identify subclinical vascular disease. Thus, a noninvasive, convenient, and efficient method should be developed to detect vascular lesions. In TCM, visceral pathological changes and other information can be obtained by detecting pulses. For instance, pathological changes in CHD are clearly reflected in pulse diagnosis information. Therefore, CHD could be considered a breakthrough point for providing a theoretical basis for investigation the TCM pulse.

Modern medical research has shown that the arterial pulse is caused by heart contraction. The left ventricle ejects blood into the aorta through the aortic valve, causing the velocity, pressure, and diameter in the arterial tree to pulsate [5]. The signal acquired from the radial artery is the comprehensive reflection of the wave form (shape), velocity (fast or slow), period (rhythm), and swing (intensity) of pulse waves in the radial artery [6]. Pulse waves contain human physiological and pathological information. Pulse diagnosis is conducted to investigate this information. Thus, pressure pulse charts, which show pulses in TCM, can be used noninvasively and conveniently to provide insight into visceral diseases, particularly cardiovascular diseases such as CHD. Subjective judgments and description of pulse in TCM rely on the experience of doctors. However, clinical application and development of this technique are restricted. Thus, further study should be conducted using various modern information-processing methods, including time domain analysis [7]; frequency domain analysis, such as the Fourier transform [8]; and combined time-frequency analysis, such as wavelet decomposition [9]. Many quantifiable parameters can be obtained; moreover, fuzzy clustering, the Bayes classifier, support vector machine, artificial neural networks, and other methods can be used to classify and recognize pulse [10–13]. For feature extraction of pulse, although the Fourier transform provides the average distribution of signal energy, this technique fails to characterize time-varying information of signal frequency and cannot describe the time domain local features of signals. For characterizing the nonstationary signals of pulse waves, an extra high-frequency harmonic signal is necessary. However, a correct and reasonable interpretation of the signal cannot be provided because the high-frequency harmonic signal is noninherent [14]. Although wavelet transform is essentially a Fourier transform with an adjustable window, the signal in a wavelet window must be stable and cannot eliminate the limitations of Fourier analysis.

Hilbert-Huang transform (HHT) is a new self-adapting time-frequency analytic method. This method can be used to conduct self-adapting time-frequency decomposition according to local time-varying characteristics and can overcome the defects of insignificant harmonic component showing nonstationary and nonlinear signal in traditional methods. Moreover, this method enables obtaining high time-frequency resolution and sufficient time-frequency aggregation; hence, this technique is suitable for nonstationary and nonlinear signal analysis [15]. HHT is composed of empirical mode decomposition (EMD) and the Hilbert transform. The core of this technique is EMD, which is performed on the basis of the time-scale feature of data. Therefore, EMD is more suitable for nonstationary and nonlinear data processing than the Fourier and wavelet methods depending on the transcendental function based on the decomposition method. We used the random forest algorithm as the classifier. The training and prediction speeds of this algorithm are high, and an internal unbiased estimation of a generalization error can be generated. The interaction between features and their degree of importance can be detected, and the over fitting does not occur. For an unbalanced classified data set, this algorithm can balance the error and can be easily parallelized. Therefore, we analyzed and determined the pulse condition of patients with CHD in this study by using EMD time series analysis method and the random forest recognition algorithm.

## 2. Clinical Material

TCM pulse refers to the pulse sensed by doctors as they palpating the examinee's radial artery with their fingers. Imitating TCM doctors, measurement equipment (cooperatively developed by our research team and Shanghai Asia-Pacific Computer Co. Ltd) was employed to acquire pulse recordings, which provided the basis for objective pulse analysis.

Pulse recordings used in this study were acquired from 342 volunteers for 60 sec with a sampling rate of 720 Hz. Two groups without respiratory system and nervous system disorders were studied. Each subject was instructed to relax for more than 5 min before pulse was recorded.

Group 1 included 225 inpatients with CHD aged 64.8 ± 10.57 years from Longhua Hospital and Shuguang Hospital, which are affiliated to Shanghai University of Traditional Chinese Medicine.

Group 2 included 117 normal subjects, who are selected as control subjects aged 52.17 ± 11.00 years. The subjects were players in the “2010 Zhangjiang ball game competition for the elderly” and staff from Shanghai University of Traditional Chinese Medicine. These subjects have no documented history of cardiovascular disorders.

## 3. Method

### 3.1. HHT

Huang et al. proposed the HHT method [16]. The proposed technique is based on the intrinsic mode function (IMF) and EMD. EMD involves decomposing a given signal from a small scale to a large scale to obtain the component signal IMF according to local characteristic time scale. The IMF obtained through decomposition must satisfy two conditions: (1) for the entire signal length, the numbers of extreme points and zero crossing of an IMF component must be equal to or differ by 1 at most.; (2) at any time, the average value of an upper envelope point defined by the maximum and a lower envelope defined by the minimum is 0.

EMD was performed as follows.

(1) Three sample interpolation fittings were used for obtaining the upper envelope curves and lower envelope curves of the signal to calculate the average value of the upper and lower envelope curves at each point and, thus, obtain the average curve *m*_1_(*t*).

(2) The average curve minus *m*_1_(*t*) was subtracted from the original signal *s*(*t*). The first component *h*_1_(*t*) was subsequently obtained.

If *h*_1_(*t*) satisfied two conditions of the IMF, then *h*_1_(*t*) was the IMF component in the first order. Otherwise, the difference value between *h*_1_(*t*) and the other envelope median value was calculated again. The IMF component *c*_1_(*t*) in the first stage can only be obtained if the difference value sequence satisfies the two conditions of the IMF.

(3) The component *c*_1_(*t*) was subtracted from the original signal to obtain the residual signal of the original signal *r*_1_(*t*). The signal *r*_1_(*t*) was redefined as the original signal. Steps (1) to (3) were repeated and *n* IMF components were obtained until *r*_*n*_(*t*) was converted into monotone function or reached a constant value.

By performing EMD,* n* IMF components and a residual signal *r*_*n*_(*t*) can be obtained. Thus, the original signal can be represented using the following equation:

(1) $$\begin{array}{c} s\left(t\right)=\overset{n}{\underset{i=1}{\sum }}c_{i}+c_{n}\left(t\right). \end{array}$$

Each IMF component *c*(*t*) and its Hilbert transform were used to construct an analytic signal as shown in the following equation:

(2) $$\begin{array}{c} z_{i}\left(t\right)=c_{i}\left(t\right)+j\tilde{c_{1}}\left(t\right)=a_{i}\left(t\right)e^{j\emptyset_{i}\left(t\right)}, \end{array}$$

where *a*_*i*_(*t*) is the amplitude function, which shows the instantaneous amplitude energy of the signal at each sampling point and *∅*_*i*_(*t*) is the phase function, which shows the instantaneous phase of a signal at each sampling point; instantaneous frequency *w*(*t*) can be obtained by calculating its derivative. Thus, the amplitude and frequency of the signal are functions of time and are plotted on the time-frequency plane to obtain the Hilbert spectra *H*(*w*, *t*). Hilbert showed the global transformation rule for a signal amplitude with time and frequency conversions; this corresponds to the distribution of signal energy in various characteristic scales (time or frequency) to a certain extent.

### 3.2. Energy Feature of a Pulse Signal Based on EMD

IMF component was obtained after a signal was decomposed through EMD. The energy of each IMF component was calculated according to the following equation:

(3) $$\begin{array}{c} E_{i}=\overset{\infty }{\underset{-\infty }{\int }}{\left|c_{i}\left(t\right)\right|}^{2}dt,\;i=1,2,\ldots ,n. \end{array}$$

For subsequent statistical analysis, normalized energy can be obtained according to the following:

(4) $$\begin{array}{c} E=\overset{n}{\underset{i}{\sum }}E_{i},\\ E_{i}^{\prime }=\frac{E_{i}}{E}. \end{array}$$

### 3.3. Sample Entropy of Pulse Signals Based on EMD

Richman and Moorman [17] developed a new and related complexity measure, sample entropy (SampEn), based on the research of Grassberger, and so forth. The sample entropy calculation of each IMF component is introduced as follows.

(1) For a time series (pulse signal) of *N* points *u*(*j*), *j* = 1,…, *N*. The time series *u*(*j*) forms a set of* m*-dimensional vectors *Xm*(*i*), *i* = 1,…, *N* − *m* + 1, where *Xm*(*i*) = {*u*(*i* + *k*)∣*k* = 0,…, *m* − 1} is the vector of *m* data points from *u*(*i*) to *u*(*i* + *m* − 1).

(2) The distance between two such vectors is defined as the maximum difference in their corresponding scalar components:

(5) $$\begin{array}{c} d\left[Xm\left(i\right),Xm\left(j\right)\right]=\max\left\{\left|u\left(i+k\right)-u\left(j+k\right)\right|\right\}, \end{array}$$

where *k* = 0, 1, …, *m* − 1.

(3) Given *r* of *Xm*(*i*), for every value from 1 to *N* − *m*, the number of *d*[*Xm*(*i*), *Xm*(*j*) < *r*] is calculated, and *j* ≠ *i* to exclude self-matches. The ratio of the number to (*N* − *m* − 1) is defined as follows:

(6) $$\begin{array}{c} B_{i}^{m}\left(r\right)=\frac{1}{N-m-1}\left\{{Num}_{d\left[X_{m}\left(i\right),X_{m}\left(j\right)\right]<r}\right\}, \end{array}$$

where 1 ≤ *j* ≤ *N* − *m*. The average value of *B*_*i*_^*m*^(*r*) is defined as follows:

(7) $$\begin{array}{c} B^{m}\left(r\right)=\frac{1}{N-m}\overset{N-m}{\underset{i=1}{\sum }}B_{i}^{m}\left(r\right). \end{array}$$

(4) Given *r* of *X*_*m*+1_(*i*), set

(8) $$\begin{array}{c} A_{i}^{m}\left(r\right)=\frac{1}{N-m-1}\left\{{Num}_{d\left[X_{m+1},X_{m+1}\left(i\right)\right]<r}\right\}, \end{array}$$

where 1 ≤ *j* ≤ *N* − *m*, and *j* ≠ *i*. The average value of *A*_*i*_^*m*^(*r*) is then obtained using the following equation:

(9) $$\begin{array}{c} A^{m}\left(r\right)=\frac{1}{N-m}\overset{N-m}{\underset{i=1}{\sum }}A_{i}^{m}\left(r\right). \end{array}$$

(5) *B* ^*m*^(*r*) is the probability that two sequences match at *m* points, whereas *A* ^*m*^(*r*) is the probability that two sequences match for *m* + 1 points. The parameter SampEn(*m*, *r*) is defined as follows:

(10) $$\begin{array}{c} \underset{N\to \infty }{\lim}\left\{-\ln\left(\frac{A^{m}\left(r\right)}{B^{m}\left(r\right)}\right)\right\}. \end{array}$$

The sample entropy of pulse signal with finite length *N* is estimated using the following statistic:

(11) $$\begin{array}{c} SampEn\left(m,r,N\right)=-\ln\left(\frac{A^{m}\left(r\right)}{B^{m}\left(r\right)}\right). \end{array}$$

The parameters *m* and *r* are used to estimate SampEn [18]. Pincus suggested that *m* = 2, *r* = 0.1 to 0.25*δ*, and *δ* is the standard deviation of the original signal *u*(*i*), *i* = 1,…, *N*. SampEn(*m* = 2, *r*, *N*) reflects the rate of information production as *m* increases from 2 to 3. The higher the SampEn value is, the higher the rate of the information production, indicating that the signal is more complex.

### 3.4. Random Forest Recognition Method

A random forest [19] is composed of numerous decision trees, which are formed using a stochastic method. Thus, it is also called a random decision tree. Trees in a random forest do not correlate. After test data are used as input in a random forest to classify each decision tree, the category with the highest classification results in all decision trees is selected as the final result. Therefore, a random forest is a classifier that contains multiple-decision trees, and its output category relies on the mode of output categories of individual trees.

A random forest resampling technique uses the bootstrap method, which entails repeatedly and randomly selecting *k* samples from the original training sample set *N* to generate new training sample sets. Subsequently, *K* classification trees are generated according to the bootstrap sample set to construct random forests. The classification results of the new data rely on the score formed by the vote of the classification tree. The algorithm is presented as follows.

(1) The original training set is *N*, and the bootstrap method is used to randomly select *K* new self-help sample sets and to construct *K* classification trees. Samples not drawn at each time constitute *K* data outside the bag.

(2) In total, *m*_all_ variables are set, and *m*_try_ variables (*m*_try_ ≪ *m*_all_) are randomly selected at each node of each tree. The variable with the greatest classification ability is selected. The variable classification threshold is determined by examining each classification point.

(3) Each tree grows to the maximum size, with on pruning.

(4) The generated multiple classification trees constitute random forests. New data are obtained and classified according to the random forest classifier. The classification results depend on the number of votes provided by the tree classifiers.

Random forests are an improvement of the decision tree algorithm, in which multiple decision trees are merged. Each tree is established on the basis of an independently extracted sample. All of the trees in the forest are uniformly distributed. Classification error depends on the classification ability of each tree and the correlation among these trees. Feature selection is performed to divide each node by using a stochastic method. The errors generated in various circumstances are then compared. Internal estimation error, or the classification and correlation ability, is detected to determine the number of selected features. The classification capability of a single tree may be low. The most likely classification of a test sample is selected after a high number of decision trees are randomly generated and after statistical analysis is performed according to the classification result of each tree.

The number of decision trees in a random forest in this study was 500, and *m*_try_ took the mean square root of *m*_all_.

## 4. Results

### 4.1. Statistical Analysis of Energy and Sample Entropy of Pulse Signal Based on EMD

**Figure 1:**
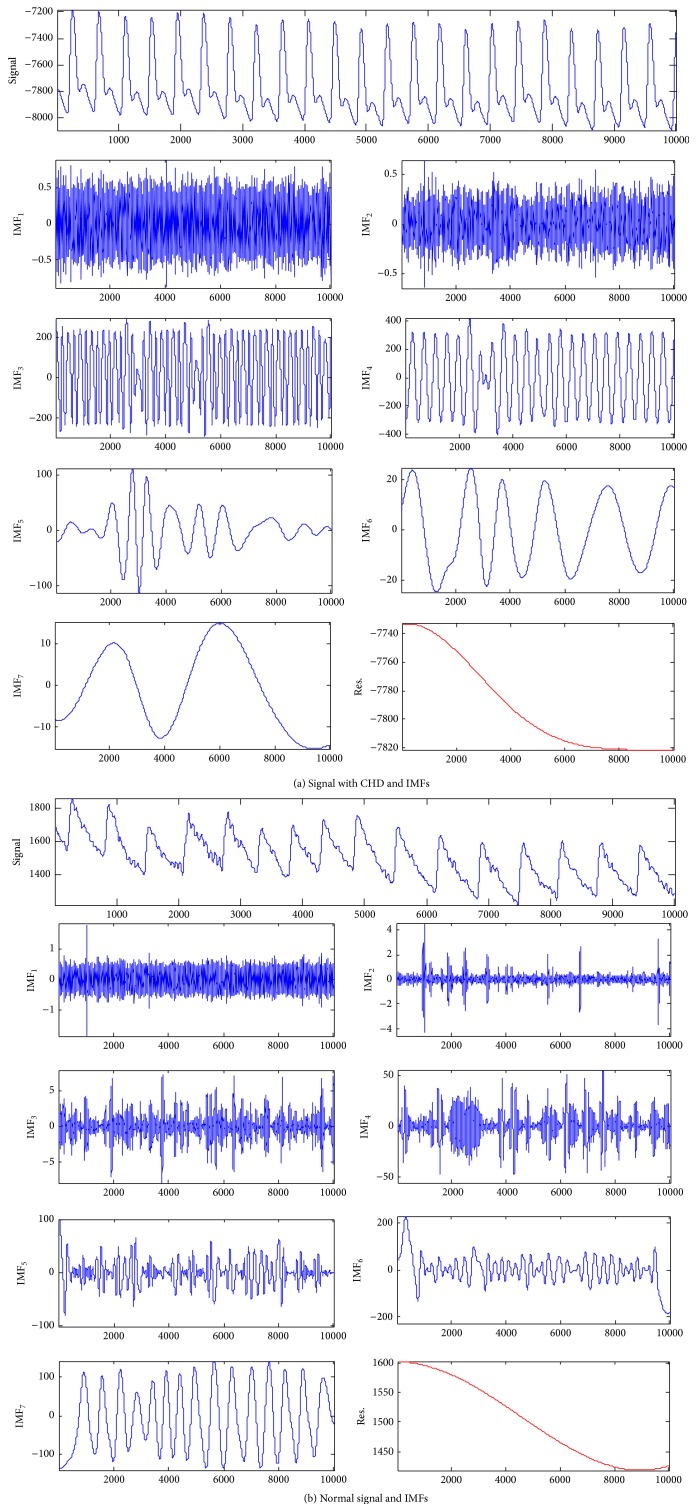
IMFs of a pulse graph for a normal subject and a patient with CHD after EMD.

EMD involves adaptively decomposing a signal frequency into a series of IMFs from a high level to a low level. IMF at each level adaptively showed signal characteristics with various resolutions. The amplitude of the IMF at each level at each time differed. The amplitude showed the strength change in the signal at a modal. Most pulse signals can be decomposed to level 7 or higher through EMD (IMF_1_–IMF_*i*_, *i* ≥ 7), and only two pulse signals of patients with CHD could be decomposed to level 6 (IMF_*i*_ = 0, *i* ≥ 7). In EMD, the modal energy at high levels was low; their effects on the entire system were weak. Therefore, we analyzed the IMF_1_–IMF_7_ in front of all modals further. The IMF at each level of a patient with CHD and one normal pulse signal after EMD are shown in Figure 1.

Figure 1 shows that the components, including IMF_1_–IMF_7_ and the residual parameters res., were obtained after EMD of the pulse signal. The frequencies of IMF_1_–IMF_7_ decreased successively, and the amplitudes of IMF_1_–IMF_7_ increased progressively. The differences of IMFs between Figures 1(a) and 1(b) can be observed. For example, Figure 1(b) shows that components of IMF_1_–IMF_7_ had higher morphological variation than those in Figure 1(a), reflecting the irregularity of a normal pulse signal, and had more high-frequency parts especially in IMF_3_–IMF_7_ than those in Figure 1(a). In order to quantitatively describe the differences between the CHD patients and the healthy subjects, we extracted the IMF energy and the IMF sample entropy of the pulse signals to make an analysis.

We observed that the variances were nonhomogeneous in the distribution of the IMF energy and the IMF sample entropy of the pulse signals in the CHD and normal groups by using IBM SPSS20.0 statistical software. Thus, we used a nonparametric test for statistical analysis. For the nonparametric test of independent samples of the two groups, we used the rank sum test method to calculate the statistical difference between the two groups.  Table 1 shows the statistical difference in the average rank of IMF energy between the two groups. The average rank with IMF normalized energy in the normal group was significantly greater than that in the CHD group.  Table 2 shows the statistical difference in the average rank of the IMF sample entropy between the two groups. Owing to the high-frequency modes IMF_1_ and IMF_2_ were caused by interference, the modes IMF_1_ and IMF_2_ were discarded without the following analysis. The intermediate-frequency modes, such as IMF_3_, IMF_4_, IMF_5_, and IMF_6_ in the CHD group were significantly lower than those in the normal group. No statistically significant difference was observed in IMF_7_ between the two groups.

### 4.2. Pulse Recognition Based on the Random Forest Classifier

We classified and recognized the energy and sample entropy characteristics of the IMFs of the two groups of pulses by using random forest classifier, and the recognition results are shown in Table 3.

Table 3 shows that the average recognition rate was 76.35%, when we used only the sample entropy of IMFs as the feature vector to recognize pulses in the two groups. Furthermore, the average recognition rate was 84%, when we used only the IMF energy as the feature vector to recognize the pulses in the two groups. If the IMF energy and IMF sample entropy were jointly used as the feature vector to recognize pulses in the two groups, the average recognition rate could reach 90.21%.

## 5. Discussion

Adaptive HHT exhibits a high time-frequency resolution and local orthogonal self-adaption; moreover, this technique is superior to the wavelet transform and other signal analysis methods. Adaptive HHT can be used to examine instantaneous frequency and energy at multiple scales and is a powerful tool for analyzing biomedical signals [20]. The IMF sequence of pulse signals obtained using EMD is directly separated from the original time series data. The number of decompositions need not be determined in advance without being affected by human factors. Thus, the IMF sequence can effectively reflect the inherent physical characteristics of original data. Its decomposition is objective, inherent, and adaptive. The IMF at each level represents band information with a specific meaning.  Table 1 shows that the IMF energy at each level in the CHD group was significantly lower than that in the normal group, which indicates that the energy of the cardiovascular system in patients with CHD was likely lower than that in the normal subjects. Human biological waves such as the pulse wave can directly reflect physical function and health status. Human biological wave energy can be produced through the metabolism of water, air, sunlight, food, and other substances, which are absorbed by molecules and cells of the human body and can be naturally supplied with nutrients and oxygen. High pulse wave energy in the healthy people may result when qi of the viscera and meridians moves harmoniously. As Table 2 shows, the sample entropy of pulse signals in patients with CHD in modes IMF_3_–IMF_6_, which showed statistically significant differences, was lower than that in the normal subjects. Entropy is the rate at which new information is generated; the entropy value reflects the complexity of a system. A higher entropy value shows that the signal generated by the system is possibly random and irregular and that the system adaptability is stronger. A lower entropy value shows that the signal generated by the system is simpler and regular, and that the system adaptability is lower. The result of Table 2 indicated that the adaptive ability of the cardiovascular system of patients with CHD was likely lower than that of the normal subjects. This finding is consistent with a relatively weaker physiological complexity of the human body in the pathological state and, thus, corresponds to regular pathological states. The energy and sample entropy of IMFs are valuable features for characterizing the pathological state of pulse in patients with CHD. The HHT method can be used to analyze pulse signals of nonstationary dynamic change. This method can sensitively capture the primary features of various pulse signal components with the dynamic changes in time and frequency. The energy and sample entropy of IMF provides a new basis for the feature extraction and pattern recognition of various pulses.

Random forest is a classifier that produces highly accurate with high training and prediction speeds. This classifier can generate an internal unbiased estimation of a generalization error during classification. Furthermore, overfitting never occurs. Random forest can also balance the errors in unbalanced data sets. In this study, we classified and determined the IMF energy and IMF sample entropy of pulse signals by using random forest classifier. The energy of IMFs, sample entropy of IMFs, and their combination were inputted as pulse feature vectors; the corresponding average recognition rates were 84%, 76.35%, and 90.21%, respectively. Compared with the separate use of IMF energy or IMF sample entropy as a feature vector, the combined use of IMF energy and IMF sample entropy as a feature vector improved the classified accuracy for the CHD group and the normal group. Although the sample size in the CHD group was differed from that in the normal group, the unbalanced sample capacity of random forest was superior. Nevertheless, we achieved satisfactory classification results.

## 6. Conclusion

Pulse diagnosis is a characteristic diagnostic method in TCM. Pulse detection is a noninvasive technique with the simple and easy operation and the stable performance, which does not require expensive equipment. A pressure pulse signal that corresponds to the pulse condition in TCM can be detected noninvasively and conveniently to obtain the pathological and physiological status of the cardiovascular system. We extracted the energy and sample entropy of IMFs of pulse signals as pulse features and used random forests as the classifier of pulse signals. The results illustrate that the proposed methods or pulse-signal processing and classification is effective and efficient. This study offers a new method for developing and promoting a noninvasive pulse diagnostic technique. Furthermore, this research provides objective and quantitative parameters of TCM pulse and lays a foundation for research on objective and quantitative criteria on disease diagnosis or Zheng differentiation.

## Figures and Tables

**Table 1:** Rank sum test of IMF energy in two groups.

	(Mean rank)						
Group	IMF_1_	IMF_2_	IMF_3_	IMF_4_	IMF_5_	IMF_6_	IMF_7_
The CHD	172.10	132.50	148.68	153.93	152.81	146.98	142.89
The normal subjects	250.65^*^	240.32^*^	209.35^*^	199.29^*^	201.45^*^	212.59^*^	220.43^*^

^*∗*^ *P* < 0.001, *versus* normal group.

**Table 2:** Rank sum test of IMF sample entropy in two groups.

	(Mean rank)						
Group	IMF_1_	IMF_2_	IMF_3_	IMF_4_	IMF_5_	IMF_6_	IMF_7_
The CHD	180.28	188.17	156.74	143.86	150.93	158.98	164.09
The normal subjects	148.86^*^	133.77^*^	193.91^*^	218.56^*^	205.04^*^	189.64^*^	179.84

^*∗*^ *P* < 0.001, *versus* normal group.

**Table 3:** Average recognition rate of different pulse characteristics obtained using random forest (%).

Group	Feature		
	IMF sample entropy	IMF entropy	Combination of IMF sample entropy and IMF sample entropy
The CHD group	88.32 ± 3.89	89.20 ± 6.76	95.49 ± 2.78
The normal group	53.48 ± 10.15	73.99 ± 15.47	80.07 ± 9.13
The two groups	76.35 ± 4.77	84.00 ± 5.04	90.21 ± 4.09

## References

[1] Wang Y., Xu A. (2014). Zheng: a systems biology approach to diagnosis and treatments. *Science*.

[2] Cheung F. (2011). TCM: made in China. *Nature*.

[3] Jones E. L., Naoumova R. P. (2004). Genetics of amilial combined hyperlipidemia and risk of coronary heart disease. *Human Molecular Genetics*.

[4] Wang H.-Y., Wang J., Liu W.-P. (2006). Application of early vascular disease detection system in patients with cardiovascular disease. *Medical Journal of Chinese People’s Health*.

[5] Nichols W. W., O’Rourke M. F. (2005). *McDonald’s Blood Flow in Arteries: Theoretic, Exprimental and Clinical Principles*.

[6] Liu Z. R. (1982). Pulse and hemodynamics in traditional Chinese medicine. *Chinese Journal of Science*.

[7] Yan H. X., Wang Y. Q., Li F. F. (2005). Research and application of pulse analysis. *Chinese Archives of Traditional Chinese Medicine*.

[8] Jiang B., Song Z. C. (2007). Spectral analysis of pulse signals. *Techniques of Automation and Application*.

[9] Chen L., Yang L.-J. (2008). Identifying pulse signals based on fractal theories and wavelet. *Aeronautical Computing Technique*.

[10] Shu J.-J., Sun Y. (2007). Developing classification indices for Chinese pulse diagnosis. *Complementary Therapies in Medicine*.

[11] Zhang W.-P., Zhang Y., Zhang S.-S. (2008). Application on wavelet and neural network in the analysis and pattern recognition of the manifestation of the pulse for detection of cerebrovascular disease. *Progress in Biomedical Engineering*.

[12] Guo Q.-L., Wang K.-Q., Zhang D.-Y., Li N.-M. A wavelet packet based pulse waveform analysis for cholecystitis and nephrotic syndrome diagnosis.

[13] Wang H.-Y., Xu S. (2009). Automatic pulse recognition method based on bayesian classifier. *Chinese Journal of Biomedical Engineering*.

[14] Zhao Z.-D., Tang X.-H., Zhao Z.-J., Pan M., Chen Y.-Q. (2005). Spectrum analysis of heart sound signal based on Hilbert-Huang transform. *Chinese Journal of Sensors and Actuators*.

[15] De-jie Y., Jun-seng C., yu Y. (2006). *Application of Hilbert-Huang Transform in Mechanical Fault Diagnosis*.

[16] Huang N. E., Shen Z., Long S. R. (1998). The empirical mode decomposition and the Hilbert spectrum for nonlinear and non-stationary time series analysis. *Proceedings of the Royal Society of London*.

[17] Richman J. S., Moorman J. R. (2000). Physiologica time-series analysis using approximate entropy and sample entropy. *The American Journal of Physiology—Heart and Circulatory Physiology*.

[18] Pincus S. M. (2001). Assessing serial irregularity and its implications for health. *Annals of the New York Academy of Sciences*.

[19] Breiman L. (2001). Random forests. *Machine Learning*.

[20] Peng C.-K. Potential HHT Applications in Biomedical Signal Analysis. http://www.fuentek.com/technologies/hht.htm.

